# Nose Bleed

**Published:** 1870-03

**Authors:** 


### NOSE BLEED,

Or "Epistaxis," in medical language, is always regarded as an unwelcome event, yet it always does good; generally prevents an attack of head-ache or more serious sickness, and sometimes arrests apoplexy and sudden death; hence no effort should be made to arrest it, until a tea-spoonful or two or more has fallen. Sometimes the nose bleeds alarmingly, and now and then persons have bled to death. When the nose threatens to bleed excessively, put the feet in hot water; if that does not arrest it, lie down and place the head in a position in which a stream of cold water can be poured upon it, without wetting the clothing. Sometimes it is almost instantly arrested by snuffing up the dust at the bottom of a tea canister. It may be stopped by applying a mustard plaster from the nape of the neck, a foot downward and a foot across. But any thing short of several teaspoonfuls should not be arrested, for nature is seeking to avert some disease.

